# SQUAMOSA PROMOTER BINDING PROTEIN-LIKE 9 and 13 repress *BLADE-ON-PETIOLE 1* and *2* directly to promote adult leaf morphology in Arabidopsis

**DOI:** 10.1093/jxb/erad017

**Published:** 2023-01-11

**Authors:** Tieqiang Hu, Darren Manuela, Mingli Xu

**Affiliations:** Department of Biological Sciences, University of South Carolina, Columbia, SC 29208, USA; Department of Biological Sciences, University of South Carolina, Columbia, SC 29208, USA; Department of Biological Sciences, University of South Carolina, Columbia, SC 29208, USA; University College Dublin, Ireland

**Keywords:** Blade, BOP1, BOP2, petiole, SPL9, SPL13, vegetative phase change

## Abstract

The juvenile-to-adult phase transition during vegetative development is a critical decision point in a plant’s life cycle. This transition is mediated by a decline in levels of miR156/157 and an increase in the activities of its direct targets, SQUAMOSA PROMOTER BINDING PROTEIN-LIKE (SPL) proteins. In Arabidopsis, the juvenile-to-adult transition is characterized by an increase in the length to width ratio of the leaf blade (a change in the distal region of a leaf), but what mediates this change in lamina shape is not known. Here, we show that ectopic expression of *SPL9* and *SPL13* produces enlarged and elongated leaves, resembling leaves from the *blade-on-petiole1* (*bop1*) *bop2* double mutant. The expression of *BOP1/BOP2* is down-regulated in successive leaves, correlating with the amount of miR156 and antagonistic to the expression of SPL9 and SPL13 in leaves. SPL9 and SPL13 bind to the promoters of *BOP1/BOP2* directly to repress their expression, resulting in delayed establishment of proliferative regions in leaves, which promotes more blade outgrowth (the distal region of a leaf) and suppresses petiole development (the proximal region of a leaf). Our results reveal a mechanism for leaf development along the proximal–distal axis, a heteroblastic character between juvenile leaves and adult leaves.

## Teaser

Repression of *BOP1/2*expression by miR156-targeted SPL transcription factors controls establishment of proliferative regions in Arabidopsis leaves, thus controlling the blade and petiole development along the proximal–distal axis.

## Introduction

After germination, flowering plants go through a juvenile vegetative phase and an adult vegetative phase before they flower. During the juvenile phase in Arabidopsis, the levels of miR156 and miR157 are high. As the shoot matures, the levels of miR156 and miR157 decline, and the activities of a group of SQUAMOSA PROMOTER BINDING PROTEIN-LIKE (SPL) transcription factors increase (Wu *et al.*, 2009; Xu *et al.*, 2016b; He *et al.*, 2018). The juvenile-to-adult phase transition (vegetative phase change) is largely controlled by the miR156–SPL module, and changes in miR156 and SPL activities resulted in changes in a plant’s immune response, plant–herbivore interactions, temperature stress responses, reproductive competence, and grain yield (Jiao *et al.*, 2010; Stief *et al.*, 2014; Mao *et al.*, 2017; Wang *et al.*, 2018; Hyun *et al.*, 2019). Therefore, vegetative phase change is essential for the survival and reproductive fitness of a plant, and it is important to understand the mechanisms of vegetative phase change and how vegetative phase change traits are regulated.

In the juvenile phase, the Arabidopsis shoot produces small round leaves without abaxial trichomes. As the miR156/157 levels decline and the activities of their direct targets (SPLs) increase, the shoot produces leaves with more serrations at the blade margin, abaxial trichomes, and a more elongated blade (a change of the distal region of a leaf). Blade margin serration is largely controlled by CUP-SHAPED COTYLEDON (CUC) and TEOSINTE BRANCHED1/CYCLOIDEA/PCF (TCP) transcription factors, with increased CUC activities inducing blade margin serration (Palatnik *et al.*, 2003; Bilsborough *et al.*, 2011; Hasson *et al.*, 2011). Increased activities of the SPL proteins (such as SPL9) in adult leaves destabilize the CUC–TCP complex by physically interacting with TCP, releasing CUC proteins to induce leaf margin serration (Rubio-Somoza *et al.*, 2014). The occurrence of abaxial trichomes is controlled by the interaction between the miR172 targeted TARGET-OF EAT1 (TOE1)/TOE2 and the adaxial/abaxial polarity regulator KANADI1 (KAN1) and their action on the trichome initiation gene *GLABRA1* (*GL1*) (Wang *et al.*, 2019; Xu *et al.*, 2019). SPL9 and SPL15 directly activate the expression of *MIR172b*, leading to the down-regulation of its direct targets, *TOE1/2* (Wu *et al.*, 2009; Hyun *et al.*, 2016). In juvenile leaves where the activities of miR156-targeted SPL proteins are low, TOE1/2 levels are high, and they physically interact with the abaxial identity protein KAN1 to repress the expression of the trichome initiation gene *GL1* to suppress trichome production on the abaxial side of the juvenile leaves. In adult leaves where the activities of miR156-targeted SPL proteins are high, TOE1/2 levels are low and *GL1* is de-repressed, resulting in production of trichomes on the abaxial side (Kerstetter *et al.*, 2001; Wang *et al.*, 2019; Xu *et al.*, 2019). Together, the spatial and temporal interaction between KAN1 and TOE1/2 controls the spatial and temporal expression of *GL1* to control the abaxial trichome production in juvenile and adult leaves. Overexpression of *MIR172B* (*35S::MIR172B*) greatly accelerates abaxial trichome production in *35S::MIR156A* (from leaf 90 to leaf 11). The lamina shape of *35S::MIR156A*, however, cannot be restored by *35S::MIR172B* (Wu *et al.*, 2009). Blade margin serration and abaxial trichome production during vegetative phase change have been well studied, but the mechanisms that regulate leaf shape during vegetative phase change have yet to be elucidated.

*BLADE-ON-PETIOLE 1* (*BOP1*) and *BOP2* genes encode the BTB-POZ domain of NPR1 subfamily transcription co-factors and have several roles during vegetative and reproductive development (Hepworth *et al.*, 2005; Norberg *et al.*, 2005; Ha *et al.*, 2007; Jun *et al.*, 2010; Khan *et al.*, 2012). *BOP1*/*2* are expressed in leaf primordia and floral primordia, in petioles of leaves, at the base of floral organs, and at the pedicel axis connecting the pedicel to the primary inflorescence (Hepworth *et al.*, 2005; Norberg *et al.*, 2005; Ha et al., 2007, 2010; Jun *et al.*, 2010; Xu *et al.*, 2010; Khan *et al.*, 2012). Correspondingly, BOP1/2 have roles in leaf patterning along the proximal–distal axis, floral meristem identity, floral organ abscission, and fruit phyllotaxy (Hepworth *et al.*, 2005; Norberg *et al.*, 2005; Xu *et al.*, 2010; Khan *et al.*, 2012). In Arabidopsis, mutations in *BOP1/2* simultaneously result in blade outgrowth on the petiole (Hepworth *et al.*, 2005; Norberg *et al.*, 2005; Ha *et al.*, 2007; Jun *et al.*, 2010). Orthologs of BOP1/2 in *Medicago truncatula* (NOOT), pea (COCH), and *Lotus japonicus* (NOOT-BOP-COCH-LIKE) promote proximal–distal patterning in leaves and floral organ patterning, similar to the roles of BOP1/2 in Arabidopsis. NOOT and NOOT-BOP-COCH-LIKE also promote identity of nodules, an organ that is found in *Medicago truncatula* and *Lotus japonicus* but not in Arabidopsis (Couzigou *et al.*, 2012; Magne *et al.*, 2018). Orthologs of BOP1/2 in rice were reported to control leaf sheath development (proximal region of a leaf, comparable to petiole in Arabidopsis) and to be repressed by the miR156-SPL module; mutations in *OsBOP1/2/3* and the miR156-SPL module resulted in changed blade to sheath ratio and blade length to whole leaf length ratio (Toriba *et al.*, 2019, 2020). The relative length of sheath (the proximal region of a leaf) has been a major heteroblastic character in rice. However, one of the major heteroblastic characters in Arabidopsis is the relative blade shape (the distal region of a leaf). Here, we analysed Arabidopsis leaf development along the proximal–distal axis spatially and temporally. We found that the more elongated blade is accompanied by suppression of petiole development and prolonged cell proliferation activities in the blade. The more elongated blade in adult leaves is probably caused by increased activities of miR156-targeted SPLs, which directly repress *BOP1*/*2* expression, leading to the suppression of petiole development and continued outgrowth of blade.

## Materials and methods

### Plant material and growth conditions

All stocks used in this study were in the Col-0 genetic background. *bop1-3 bop2-1*, *BOP1::GUS*, and *BOP2::GUS* were gifts from Dr S. R. Hepworth (Xu *et al.*, 2010). *SPL9::sSPL9-GUS*, *SPL13::sSPL13-GUS*, *SPL9::rSPL9-GUS*, and *SPL13::rSPL13-GUS* were gifts from Dr R. S. Poethig (Xu *et al.*, 2016b). miR156-resistant *SPL13* (*rSPL13*), *rSPL13-HA*, *rSPL9-HA*, and *rSPL9-GFP* were constructed in the Golden Gate system (Engler *et al.*, 2014). Primers for making the constructs with the Golden Gate system are listed in Supplementary Table S1. The constructs were transformed into Arabidopsis Col-0 or *spl9 spl13* using the floral dipping method (Clough and Bent, 1998). Primers for genotyping *bop1*, *bop2*, *spl2*, *spl9*, *spl10*, *spl11*, *spl13*, and *spl15* were described previously (Xu *et al.*, 2010, Xu *et al.*, 2016b). Seeds were sown on Sunshine #8 potting soil, stratified at 4 °C for 2–4 d, and then transferred into Conviron growth chambers maintained at constant 22 °C in either long days (LDs; 16 h light: 8 h dark) or short days (SDs; 10 h light:14 h dark). Unless otherwise specified, all gene expression and chromatin immunoprecipitation (ChIP) analyses were performed with plants grown in LDs. Leaf length and width were measured by ImageJ software (imagej.nih.gov).

### RT–quantitative PCR

Twelve-day-old seedlings of Col-0, *spl9/13*, *rSPL13-GUS*, *rSPL13* Col-0, and *rSPL13 spl9/13* plants or specific leaves as indicated in each figure, were harvested in liquid nitrogen. Tissues were ground into fine powder in liquid nitrogen, and total RNA was extracted using TRIzol (Thermo Fisher Scientific) followed by Turbo DNase (Thermo Fisher Scientific) treatment, according to the manufacturer’s instructions. cDNA was reverse transcribed from 1 μg of RNA with SuperScript III reverse transcriptase (Thermo Fisher Scientific), and quantitative PCR (qPCR) was performed using a Bio-Rad CFX96 real-time system. Primers used for qPCR are listed in Supplementary Table S1. All RT-qPCR experiments were performed in three biological replicates, and two reference genes, *ACTIN2* (ACT2, AT3G18780) and *EUKARYOTIC TRANSLATION INITIATION FACTOR 4A1* (*EIF4A1*, AT3G13920), were used in quantification analysis.

### β-Glucuronidase staining analysis

Seedlings at different developmental stages as indicated in figures were pre-fixed in 90% acetone on ice for 10–20 min, followed by washing in β-glucuronidase (GUS) washing buffer (100 mM potassium phosphate buffer, pH 7.0, with 2 mM potassium ferricyanide, 2 mM potassium ferrocyanide, and 0.2% Triton X-100), then exchanged in GUS staining buffer. The tissue was vacuumed for 10 min and then incubated in GUS staining solution (100 mM potassium phosphate buffer, pH 7.0, with 2 mM potassium ferricyanide, 2 mM potassium ferrocyanide, 0.2% Triton X-100, and 2 mM X-Gluc) at 37 °C overnight. The chlorophyll was removed by washing stained tissues with 70% ethanol three times.

### Chromatin immunoprecipitation

Three grams of 2-week-old Col-0 or transgenic plants grown in LDs were harvested and cross-linked in 1% formaldehyde under vacuum for 15 min. Tissues were ground in liquid nitrogen and suspended in extraction buffer 1 (0.4 M sucrose, 10 mM Tris–HCl, pH 8.0, 10 mM MgCl_2_, 5 mM β-mercaptoethanol, 1 mM phenylmethylsulfonyl fluoride (PMSF), and 0.1% Triton X-100). Pellets were washed with extraction buffer 2 (0.25 M sucrose, 10 mM Tris–HCl, pH 8.0, 10 mM MgCl_2_, 5mM β-mercaptoethanol, 1 mM PMSF, and 1% TritonX-100), and resuspended in nuclei lysis buffer (50 mM Tris–HCl, pH 8.0, 10 mM EDTA, and 1% SDS). DNA was then diluted in buffer (1.2 mM EDTA, 16.7 mM Tris–HCl, pH 8.0, 167 mM NaCl, and 0.01% SDS) and sonicated using a Covaris ultrasonicator M220. The hemagglutinin (HA) antibody and green fluorescent protein (GFP) antibody used in this study were from Sigma (cat. no. 11666606001) and Thermo Fisher Scientific (cat. no. A11122), respectively. The transposon *TA3* was used as a negative control for non-specific binding. Primers used in ChIP analysis are listed in Supplementary Table S1.

## Results

### SPL13 promotes vegetative phase change and delays petiole development

**Fig. 1.**
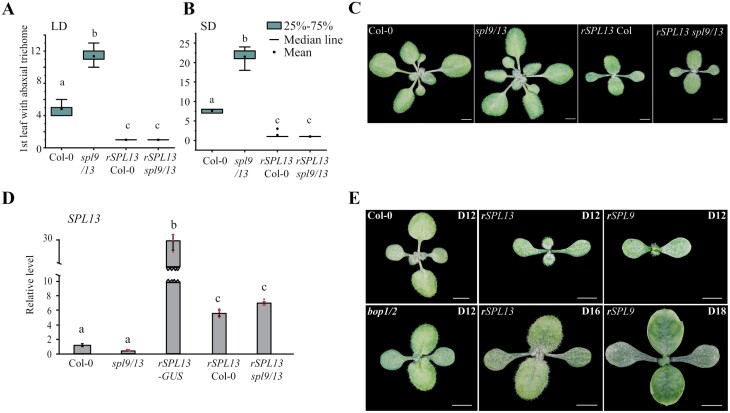
Plants ectopically expressing SPL13 and SPL9 are accelerated in vegetative phase change and mimic *bop1 bop2* double mutant at seedling stage. (A, B) Ectopic expression of *SPL13* in Col-0 and *spl9/13* accelerated abaxial trichome production in both LDs (A) and SDs (B). (C) Sixteen-day-old Col-0, *spl9 spl13*, and *rSPL13* plants in Col-0 or *spl9 spl13* (*spl9/13*) plants growing in LDs. Scale bars: 3 mm. (D) RT-qPCR analysis of *SPL13* transcripts in 12-day-old Col-0, *spl9/13*, *rSPL13-GUS*, *rSPL13* Col-0, and *rSPL13 spl9/13* seedlings. *SPL13* is highly expressed in *rSPL13-GUS*, *rSPL13* Col-0 and *rSPL13 spl9/13* seedlings. Values are relative to Col-0 and represent the mean ±SEM from three biological replicates (red dots). Shared letters indicate not significantly different groups, different letters indicate significantly different groups; *P*<0.001, one-way ANOVA. (E) Twelve-day-old Col-0, *rSPL13* plants, *rSPL9* plants, and *bop1 bop2* (*bop1/2*), 16-day-old *rSPL13* plants, and 18-day-old *rSPL9* plants growing in LDs. Note that the 16-day-old *rSPL13* and 18-day-old *rSPL9* plants mimic 12-day-old *bop1/2*. Scale bars: 3 mm.

Genetic and molecular analysis have shown that miR156 and miR157 function redundantly to repress a group of *SPL* transcription factors that promote the adult vegetative phase and flowering (Wang *et al.*, 2009; Xu *et al.*, 2016b; He *et al.*, 2018; Hyun *et al.*, 2019). Among the 10 miR156/157-targeted SPLs, SPL13 plays essential roles in promoting phase transitions (Xu *et al.*, 2016b). To further investigate the role of SPL13 in plant development, we made a construct containing a miR156-resistant *SPL13* (*SPL13::rSPL13*) and transformed this construct into both Columbia wild type (Col-0) and the *spl9 spl13* double mutant. Fourteen out of 18 (77.8%) T_1_ plants in a Col-0 background produced abaxial trichomes earlier than Col-0, whereas 18 out of 26 (69.2%) transgenics in an *spl9 spl13* background produced abaxial trichomes earlier than *spl9 spl13*. We selected a representative line from each group and analysed its phenotype and the expression level of *SPL13*. Col-0 produced abaxial trichomes on leaf 4.8 ± 0.8 in LDs and 7.6 ± 0.5 in SDs, whereas the *spl9/l3* double mutant produced abaxial trichomes on leaf 11.4 ± 0.9 in LDs and 21.5 ± 1.8 in SDs. In contrast, *SPL13::rSPL13* Col-0 and *SPL13::rSPL13 spl9/13* produced abaxial trichomes on leaf 1 ± 0.0 in LDs and leaf 1.3 ± 0.7 in SDs (Fig. 1A, B). This indicates that *SPL13::rSPL13* complements the mutant phenotype of *spl9/13*, but also suggests that *rSPL13* is overexpressed in both genetic backgrounds.

Leaf emergence was delayed in both *SPL13::rSPL13* Col-0 and *SPL13::rSPL13 spl9/13* plants. At day 16, when petioles were clearly visible in Col-0 and *spl9/13*, leaf 1 and leaf 2 of the transgenic lines did not have visible petioles (Fig. 1C), resembling the *bop1 bop2* (*bop1/2*) double mutant (Hepworth *et al.*, 2005; Norberg *et al.*, 2005; Ha *et al.*, 2007; Xu *et al.*, 2010). We grew the *SPL13::rSPL13 spl9/13* plant (*rSPL13* hereafter), *spl9/13*, *bop1/2*, and *rSPL13-GUS* plants together with Col-0 to examine their development (Supplementary Fig. S1). The *spl9/13* double mutant had a faster rate of leaf initiation than Col-0, while *rSPL13* plants, *rSPL13-GUS* plants, and *bop1/2* had a slower rate of leaf initiation than Col-0 (Supplementary Fig. S1A, B). Similarly, the *spl9/13* double mutant was delayed in producing abaxial trichomes, while the *rSPL13* and *rSPL13-GUS* plants were markedly accelerated in producing abaxial trichomes, and *bop1/2* was slightly accelerated in producing abaxial trichomes (Supplementary Fig. S1C, D). Because the phenotypes of *rSPL13* plants were more severe than *rSPL13-GUS*, we examined the relative abundance of *SPL13* in these plants by RT-qPCR. These results showed that *SPL13* transcripts were elevated 5.7-fold in *rSPL13* Col-0 and 7.1-fold in *rSPL13 spl9/13* and were elevated about 30-fold in *rSPL13-GUS* plants (Fig. 1D). This suggests that the GUS tag at the 3ʹ end interferes with the activity of SPL13.

To confirm that the less severe phenotype in *rSPL13-GUS* plants is caused by the relatively large GUS tag, we transformed *SPL13::rSPL13-HA* and *SPL9::rSPL9-HA* constructs into the *spl9/13* double mutant. The *SPL13::rSPL13-HA spl9/13* plants looked very much like the *SPL13::rSPL13 spl9/13* plants, while *SPL9::rSPL9-HA spl9/13* plants had enlarged leaves 1 and 2 in which the boundary between blade and petiole was indistinct (Fig. 1E). Like the *rSPL13* plants, the *SPL9::rSPL9-HA spl9/13* (*rSPL9* hereafter) plants produced abaxial trichomes on leaf 1, significantly earlier than wild type (WT) (5.4 ± 0.5) and *bop1/2* (4.4 ± 0.5) (Supplementary Fig. S1E). These results suggest that having a small HA tag at the C-terminus of a protein does not interfere with the activities of the protein significantly, and ectopic expression of either SPL9 or SPL13 protein suppresses petiole development.

### BOP1 and 2 act redundantly in vegetative phase change

BOP1 and BOP2 have been reported to function redundantly in regulating leaf and flower development while BOP2 functions by itself to promote photo-morphogenesis (Norberg *et al.*, 2005; Ha *et al.*, 2007; Jun *et al.*, 2010; Xu *et al.*, 2010; Khan *et al.*, 2012; Zhang *et al.*, 2017). To determine if BOP1 and BOP2 function redundantly in vegetative phase change, we examined the *bop1* and *bop2* single mutants as well as the *bop1/2* double mutant. The *bop1* and *bop2* single mutants did not have a significant effect on two major vegetative phase change characters: the first leaf with abaxial trichome and the leaf blade length: blade width ratio. However, the *bop1/2* double mutant was slightly accelerated in abaxial trichome production and had a larger blade length: blade width ratio (Supplementary Fig. S2A, B), indicating functional redundancy between BOP1 and BOP2 during vegetative phase change. The early abaxial trichome production in *rSPL9* and *rSPL13* plants could be attributed to the down-regulation of *TOE1/2* genes, which then de-repress the trichome initiation gene *GL1* (Wu *et al.*, 2009; Wang *et al.*, 2019; Xu *et al.*, 2019). Our RT-qPCR analysis of *bop1/2* leaves 1 and 4 showed that *TOE1* and *TOE2* were down-regulated in leaf 4 of the *bop1/2* double mutant, suggesting that the accelerated trichome production in *bop1/2* is probably caused by the down-regulation of *TOE1/2* (Supplementary Fig. S2C, D) (Khan *et al.*, 2015).

### Petiole development is suppressed in *rSPL13*, *rSPL9*, and *bop1/2* double mutant plants

**Fig. 2.**
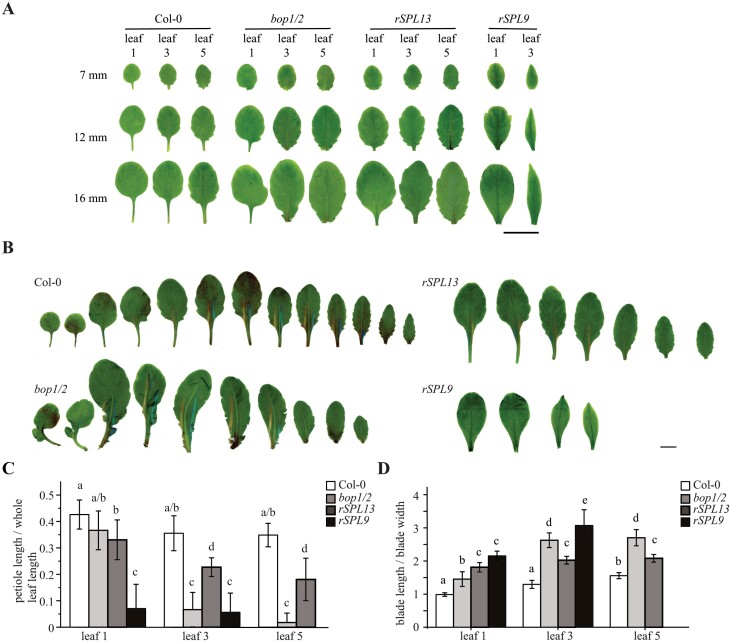
BOP1/2 promote while SPL9 and SPL13 suppress petiole development. (A) Morphology of Col-0, *bop1/2*, *rSPL13*, and *rSPL9* leaf 1, leaf 3, and leaf 5 when they are about 7, 12, and 16 mm long. (B) Heteroblasty of Col-0, *bop1/2*, *rSPL13*, and *rSPL9* plants. Plants were grown in LDs and leaves were dissected when inflorescence is about 1 cm long. (C, D) Petiole length: whole leaf length ratio (C) and blade length: blade width ratio (D) in leaves 1, 3, and 5 of Col-0, *bop1/2*, *rSPL13*, and *rSPL9* plants. Shared letters above groups indicate not significantly different groups, and different letters above groups indicate significantly different groups; *P*<0.05, two-way ANOVA. Scale bars in (A, B): 1 cm.

Although the *bop1/2* double mutant did not produce abaxial trichomes as early as *rSPL9* plants and *rSPL13* plants, the leaves of all three genotypes were very much delayed in developing a distinct petiole (Fig. 1E). We then compared leaf development in these genotypes, with a focus on petiole development. In LD conditions when the leaves are about 7 mm long, Col-0 has developed a visible and distinct petiole in leaf 1 and leaf 3 (juvenile leaves), and the petiole is just about to become visible in leaf 5 (normally an adult leaf) (Fig. 2A; Supplementary Fig. S3). However, in *bop1/2*, *rSPL13*, and *rSPL9* plants, the ­petiole was not visible in 7 mm-long primordia of leaf 1, 3, or 5 (Fig. 2A; Supplementary Fig. S3A). This suggests that BOP1/2 promote petiole development while SPL9 and SPL13 suppress petiole development. When leaf 3 and leaf 5 are about 12 mm long, Col-0 has developed distinct petioles while *bop1/2*, *rSPL13*, and *rSPL9* plants had not developed or just began to develop the petiole (Fig. 2A; Supplementary Fig. S3B). When leaf 5 was about 16 mm long, the petioles of Col-0 had elongated substantially, while *bop1/2* has not yet developed any petioles, and *rSPL13* plants had just begun to develop petioles (Fig. 2A; Supplementary Fig. S3C). Next, we examined petiole and blade development in mature leaves (when the inflorescence was about 1 cm long, and rosette leaves did not obviously grow). Overall, leaves 1 and 2 in *bop1/2*, *rSPL13*, and *rSPL9* plants were bigger than Col-0 (Fig. 2B). Studies in rice showed that leaf sheath development is disrupted and the sheath length: whole leaf length ratio is shifted in *Osbop* mutants and mSPL14 plants (Toriba *et al.*, 2019). As the examination of leaf development showed that petiole development was suppressed in *bop1/2*, *rSPL13*, and *rSPL9* plants, we examined the petiole length: whole leaf length ratio in leaf 1, leaf 3, and leaf 5 (*rSPL9* plants did not produce more than four leaves most of the time). Our results showed that the petiole length: whole leaf length ratio in *bop1/2*, *rSPL13*, and *rSPL9* plants was significantly smaller than corresponding leaves in Col-0, except for leaf 1 in *bop1/2* (Fig. 2C). As the blade length: blade width ratio (lamina shape) is one of the key characteristic traits of adult leaves in Arabidopsis, we analysed the lamina shape as well. Our data showed that the blade length: blade width ratio was significantly higher in all the corresponding leaves of *bop1/2*, *rSPL13*, and *rSPL9* plants (Fig. 2D), suggesting a more elongated lamina. Together, our results suggest that petiole development and blade development are associated: BOP1/2 suppress and SPL9/13 promote blade development, while BOP1/2 promote and SPL9/13 suppress petiole development in Arabidopsis.

### SPL9 and SPL13 repress *BOP1* and *BOP2*

**Fig. 3.**
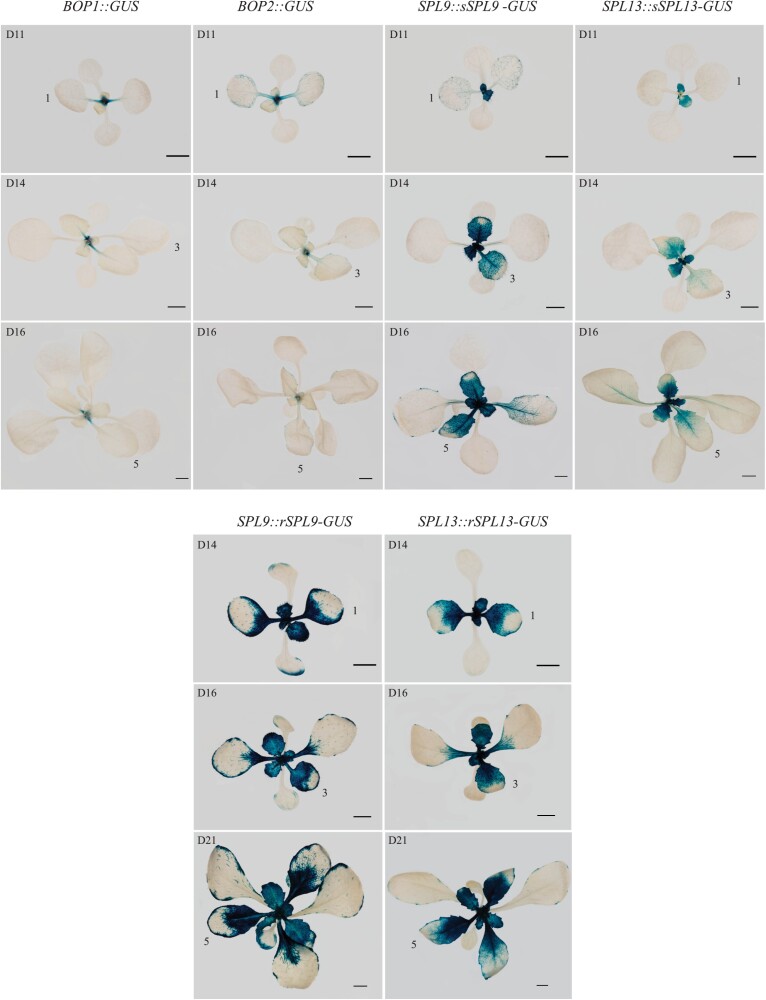
Expression of *BOP1::GUS*, *BOP2::GUS*, *SPL9::sSPL9-GUS* (sensitive to miR156), *SPL13::sSPL13-GUS*, *SPL9::rSPL9-GUS* (resistant to miR156), and *SPL13::rSPL13-GUS*. Plants were grown in LDs. All transgenic plants were in Col-0 background, and plants were harvested for GUS staining analysis when their leaf 1, leaf 3, and leaf 5 were at similar developmental stages. Scale bars: 2 mm.

The results described above suggest that BOP1/2 and SPL9/13 function in opposite directions to regulate blade and petiole development. To investigate if they act antagonistically, we compared the expression pattern of *BOP1*, *BOP2*, miR156-sensitive *SPL9* (*sSPL9*), miR156-resistant *SPL9* (*rSPL9*), *sSPL13*, and *rSPL13* genes using their GUS reporters (Fig. 3). In LDs, GUS expression driven by the *BOP1* promoter (*BOP1::GUS*) was mainly detected in the petiole, whereas *BOP2::GUS* was detected in the petiole, midvein, and veins within the lamina of leaf 1 and 2 (Fig. 3). The expression of *BOP1/2* was lower in the petiole (*BOP1*) or both the petiole and the blade (*BOP2*) of leaf 3 and 5 compared with leaf 1 and 2 (Fig. 3). sSPL9 protein detected from the *SPL9::sSPL9-GUS* plants was slightly expressed in leaf 1 and 2, whereas sSPL13 protein was excluded from leaf 1 and 2 (Fig. 3). Consistent with previous observations (Xu *et al.*, 2016b; He *et al.*, 2018), sSPL9 and sSPL13 proteins were expressed at higher levels in developing leaf 5 and 6 and were expressed at relatively lower levels in leaf 3 and 4, with the highest expression in the proximal region of the blade and petiole (Fig. 3). rSPL9 and rSPL13 proteins were highly expressed throughout the leaf when leaves were small, when petioles were not visible or just visible. They were localized to the proximal region of the blade and the petiole in larger leaves, when petioles were well developed (Fig. 3). Thus, *BOP1/2* are highly expressed in the petiole of leaf 1 and 2, whereas SPL9 and SPL13 proteins are barely expressed there. In contrast, SPL9 and SPL13 proteins are more highly expressed in the petiole of leaf 5 than *BOP1/2*, suggesting that BOP1/2 and SPL9/13 interact antagonistically.

**Fig. 4.**
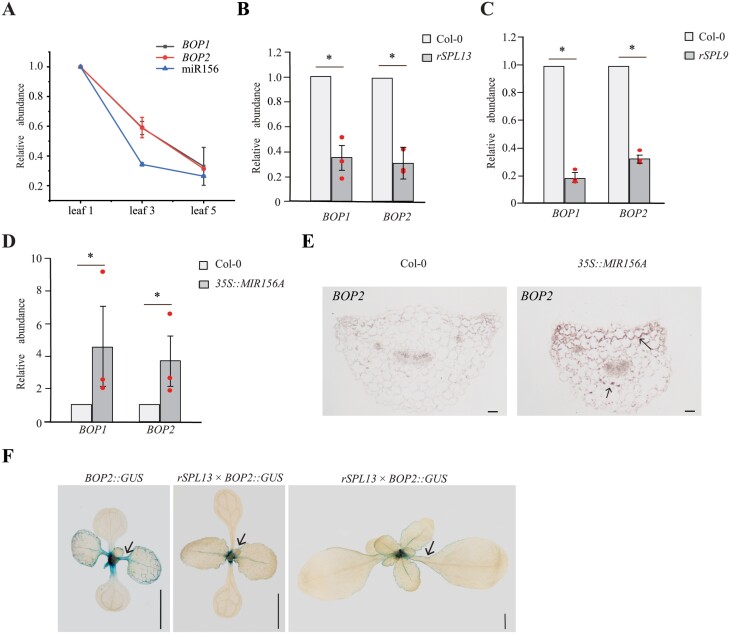
SPL9 and SPL13 repress *BOP1/2* in leaves. (A) RT-qPCR analysis of *BOP1*, *BOP2*, and miR156 in leaf 1, leaf 3, and leaf 5 in Col-0 wild-type. The amount of transcript of *BOP1*, *BOP2* and miR156 in leaf 1 was set to 1. (B) RT-qPCR analysis of *BOP1* and *BOP2* in leaf 1 and 2 of Col-0 and *rSPL13* plants. (C) RT-qPCR analysis of *BOP1* and *BOP2* in leaf 1 and 2 of Col-0 and *rSPL9* plants. (D) RT-qPCR analysis of *BOP1* and *BOP2* in leaf 5 of Col-0 and *35S::MIR156A*. The transcripts of *BOP1* or *BOP2* in Col-0 were set to 1. Values are means ±SEM from three biological replicates (red dots). *Significant difference between Col-0 and transgenic plants; *P*<0.001, one-way ANOVA. (E) *In situ* hybridization analysis of *BOP2* in the fifth leaf petiole of Col-0 and *35S::MIR156A*. *BOP2* is not expressed in the fifth leaf petiole of Col-0, but is expressed in the cortical cells underneath the adaxial epidermis and in a few cortical cells around the vascular bundle in *35S::MIR156A*. Arrows indicate expression of *BOP2*. Scale bars: 50 μm. (F) Expression of *BOP2::GUS* in Col-0 and *rSPL13* plants. Note that *BOP2* was strongly expressed in the petiole and veins of Col-0 leaf 1 and 2, while *BOP2* expression is reduced in *rSPL13* veins when its total leaf length is the same as the Col-0 (petiole was not yet visible), and *BOP2* is expressed at much lower levels at the margin of petiole when *rSPL13*’s petiole is visible. Arrows indicate expression of *BOP2*. Scale bars: 3 mm.

To confirm these observations, we measured the abundance of these transcripts by RT-qPCR (Fig. 4A). The amount of mature miR156 decreased about 70% from leaf 1 to leaf 3, whereas transcripts of *BOP1* and *BOP2* decreased about 40%. The amount of miR156 continued to drop in leaf 5, as did the transcripts of *BOP1/2* (Fig. 4A). Thus, miR156 levels are positively correlated with the transcripts of *BOP1/2*, possibly through the repression of *BOP1/2* by the SPL proteins targeted by miR156. To test this hypothesis, we examined the expression of *BOP1/2* in the first two leaves of *rSPL13* plants and *rSPL9* plants. *BOP1*/*2* mRNA levels were reduced about 60% in *rSPL13* plants and 70% in *rSPL9* plants (Fig. 4B, C). Conversely, *BOP1*/*2* mRNA levels were elevated in *35S::MIR156A* leaf 5 (Fig. 4D). To examine how miR156-targeted SPL proteins regulate *BOP2* expression we used *in situ* hybridization and reporter genes to examine the effect of SPL proteins on the expression of *BOP2*. *BOP2* was not detected in the petiole of Col-0 leaf 5 by *in situ* hybridization but was detected in the cortical cells underneath the upper epidermis and in a few cells surrounding the vascular bundle of *35S::MIR156A* leaf 5 (Fig. 4E). *BOP2::GUS* was expressed at high levels in the petiole and veins of the first two leaves of Col-0 but was expressed at a much lower level in the petioles of the first two leaves in *rSPL13* plants (Fig. 4F). Together, the RT-qPCR analysis and expression analysis suggest that SPL9 and SPL13 repress *BOP1/2* to prolong blade development and delay petiole development in Arabidopsis.

### Genetic interaction between miR156-targeted *SPL*s and *BOP1/2*

**Fig. 5.**
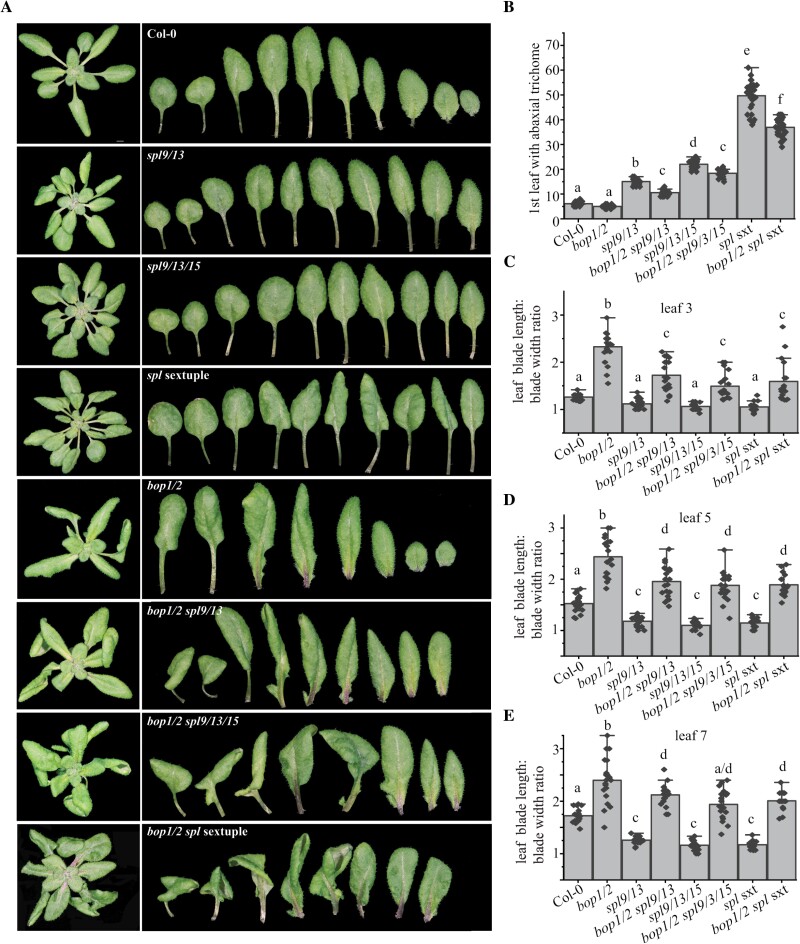
Genetic interaction between *bop1/2* and *spl* mutants. (A) Whole plant and heteroblasty of Col-0, *spl9/13*, *bop1/2 spl9/13*, *spl9/13/15*, *bop1/2 spl9/13/15*, *spl2/9/10/11/13/15* (*spl* sextuple), and *bop1/2 spl* sextuple. (B) The first leaf with abaxial trichome in Col-0, *bop1/2*, *spl* mutants, and *bop1/2 spl* mutants. (C–E) The blade length/width ratio in leaf 3 (C), leaf 5 (D), and leaf 7 (E) of Col-0, *bop1/2*, *spl* mutants, and *bop1/2 spl* mutants. Shared letters above groups indicate no significant difference between them, while different letters above groups indicate the groups that are significantly different; *P*<0.01 (B–E), one-way ANOVA.

To investigate the genetic interaction between *BOP1/2* and miR156-targeted SPLs, we crossed *bop1/2* to loss-of-function *spl* mutants. Our previous analysis showed that SPL9 and SPL13 have the most significant effect on vegetative phase change, although SPL2, SPL10, SPL11, and SPL15 also contribute to this process (Xu *et al.*, 2016b). We next introduced *bop1/2* into *spl9/13*, *spl9/13/15*, and *spl2/9/10/11/13/15* (*spl* sextuple, *spl* sxt) mutants. The rosettes of the *bop1/2 spl9/13*, *bop1/2 spl9/13/15*, and *bop1/2 spl* sextuple (*bop1/2 spl* sxt) mutants look like the *bop1/2* rosette (Fig. 5A). Their abaxial trichome production was intermediate between *bop1/2* and the corresponding *spl9/13*, *spl9/13/15*, and the *spl* sxt mutants, but much closer, respectively, to the trichome production in *spl9/13*, *spl9/13/15*, and the *spl* sxt than to *bop1/2* (Fig. 5B). Quantitative examination of the lamina shape in leaf 3, leaf 5, and leaf 7 of the mutants showed that leaves of *bop1/2* have more elongated lamina than Col-0, while *spl9/13*, *spl9/13/15*, and the *spl* sxt have a rounder lamina than Col-0 (Fig. 5C–E). The lamina shape in *bop1/2 spl9/13/15*, and *bop1/2 spl* sxt mutants was intermediate between their *bop1/2* and *spl9/13/15* or *spl* sxt parents, but much closer to the lamina shape in *bop1/2* (Fig. 5C–E). Together, these results suggest that BOP1/2 and additional targets of the miR156-targeted SPLs are involved in determining lamina shape in Arabidopsis. The abaxial trichome production, on the other hand, is largely controlled by factors other than *BOP1/*2 (Wu *et al.*, 2009; Wang *et al.*, 2019; Xu *et al.*, 2019).

### SPL9 and SPL13 binds to *BOP1* and *BOP2* directly

**Fig. 6.**
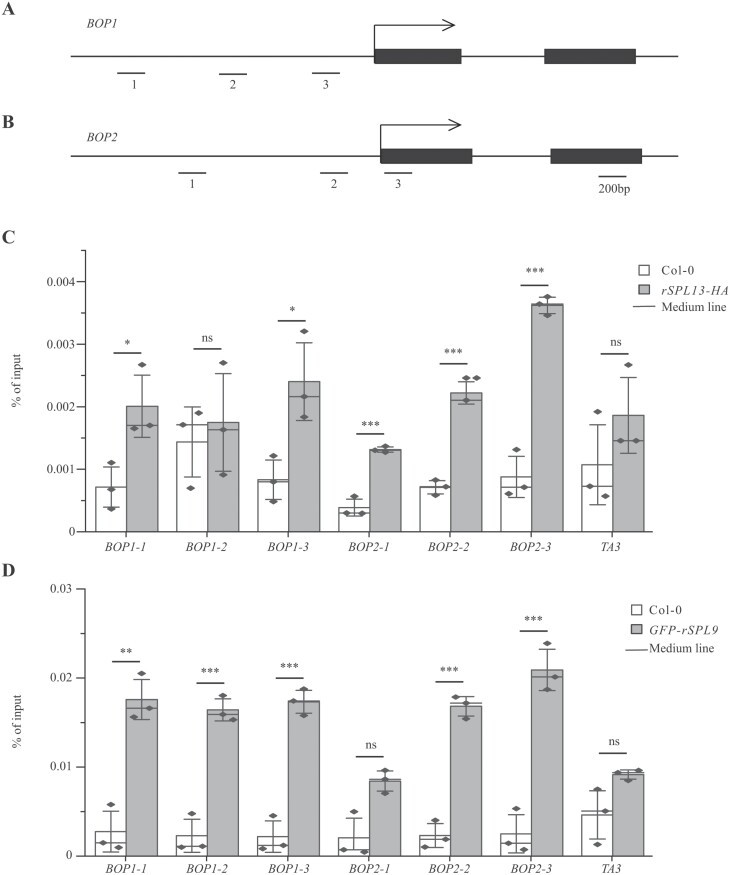
SPL9 and SPL13 bind to *BOP1* and *BOP2* directly. (A, B) Schematic diagram of the genomic structure of *BOP1* (A) and *BOP2* (B). Black boxes indicate exons, arrows indicate transcription start site. 1, 2, and 3 represent potential SPL binding sites to *BOP1/2*. (C) ChIP-qPCR analysis of rSPL13-HA occupancy at *BOP1* and *BOP2*. (D) ChIP-qPCR analysis of GFP–rSPL9 occupancy at *BOP1* and *BOP2*. Values are means ±SEM from three biological replicates (black dots). *P<0.05, **P<0.01, ***P<0.001; ns denotes not significantly different, *P*>0.05; one-way ANOVA. *TA3* is a negative control.

To investigate if miR156-targeted SPL9 and SPL13 represses *BOP1/2* directly, we used chromatin immunoprecipitation followed by qPCR (ChIP-qPCR) to examine the binding of SPL9 and SPL13 to *BOP1* and *BOP2*. We used a homozygous line from *SPL13::rSPL13-HA spl9/13* (*rSPL13-HA* hereafter) transgenic plants, and made new *SPL9::GFP-rSPL9 spl9/13* transgenic plants as the *SPL9::rSPL9-HA spl9/13* we made earlier (Fig. 1) could not be maintained as homozygotes. We chose a *SPL9::GFP-rSPL9 spl9/13* line (*GFP-rSPL9* hereafter) that produces abaxial trichomes on leaf 1(Supplementary Fig. S4), similar to the *rSPL9-HA* plants we generated previously (Supplementary Fig. S1E). We collected vegetative tissues for ChIP-qPCR analysis. We identified potential SPL binding sites in *BOP1* and *BOP2* using ATHAMAP (athamap.de) and examined the abundance of these sites in chromatin immunoprecipitated with antibodies to HA and GFP. We found that both SPL9 and SPL13 proteins bind to the *BOP1* promoter 1200–1400bp (*BOP1-1*) upstream of its transcription start site (TSS) and near its TSS (*BOP1-3*) (Fig. 6). SPL9 and SPL13 proteins bind to *BOP2* promoter and right after its TSS (*BOP2-2* and *BOP2-3*). SPL9 and SPL13 proteins also have unique binding sites at *BOP1* and *BOP2*: SPL9 binds to *BOP1-2* whereas SPL13 does not bind to this site, and SPL13 associates with *BOP2-1* whereas SPL9 does not (Fig. 6). Together, our ChIP-qPCR analysis suggests that SPL9 and SPL13 proteins have overlapping and distinctive binding sites in *BOP1* and *BOP2* genomic DNA, which may explain the similar and distinctive phenotypes of *rSPL9* and *rSPL13* plants.

### AN3 activity is prolonged in leaves of *bop1/2* and *rSPL13*

**Fig. 7.**
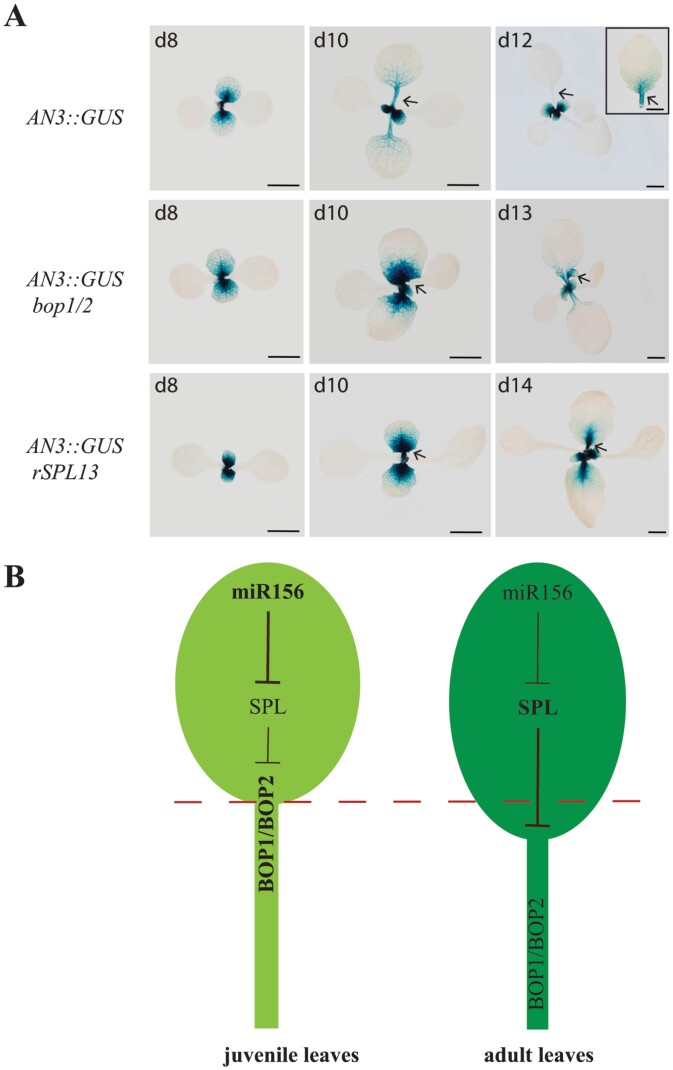
Mechanism for leaf blade development. (A) Expression of *AN3::GUS* in Col-0 and mutants. Top: expression of *AN3::GUS* in Col-0 at day (d) 8, d10, and d12 and the expression of *AN3::GUS* in leaf 5 (inset in image of d12) when it is at similar length to leaf 1 at d12. Middle: expression of *AN3::GUS* in *bop1/2* at similar developmental stages to Col-0. Bottom: expression of *AN3::GUS* in *rSPL13* plants at similar developmental stages to Col-0. Scale bars: 2 mm. Arrows indicate the expression of *AN3::GUS* in Col-0, *bop1/2* double mutant, and *rSPL13* plants at the proximal region of the blade. (B) Model for juvenile leaf and adult leaf development. In juvenile leaves where miR156 levels are high, miR156-targeted SPL levels are low, and *BOP1/2* are activated, resulting in early establishment of the blade–petiole boundary and shorter blades. In adult leaves where miR156 levels are down-regulated, miR156-targeted SPLs are up-regulated and *BOP1/2* are down-regulated, resulting in delayed establishment of the blade–petiole boundary and longer blades.

Next, we investigated the basis for the increased blade outgrowth and suppressed development of the petiole in *bop1/2*, *rSPL9*, and *rSPL13* plants. Studies of Arabidopsis leaf development suggest that establishment of a proliferative region at the junction between blade and petiole is important for blade and petiole development (Ichihashi *et al.*, 2011). This proliferative region is highly marked by the cell division marker *pCYCB1;1::Dbox::GUS* and *AN3::GUS* (Ichihashi *et al.*, 2011). Leaves of the *angustifolia3-4* (*an3-4*) mutant are smaller than WT, and the epidermal cell number in *an3-4* is proportionally smaller than WT, suggesting that AN3 regulates cell proliferation in leaves (Kawade *et al.*, 2013). Analysis of *AN3* expression in developing leaves using an *AN3::GUS* reporter showed that *AN3* is widely expressed in developing leaf primordia, is localized to the proliferative region when the blade-petiole boundary is just established, and its expression is almost gone when the leaf matures (Ichihashi *et al.*, 2011). To investigate if the establishment of the proliferative region is affected in *bop1/2* and *rSPL13* leaves, we amplified a 4-kb *AN3* promoter and fused it to GUS and transformed this construct into Col-0. We selected a line that showed a similar expression pattern to the one examined by Ichihashi *et al*. and crossed this line to the *bop1/2* double mutant and the *rSPL13* transgenic line. As leaf initiation is severely delayed in *rSPL13* homozygotes (Fig. 1B), we selected plants that were homozygous for *AN3::GUS* and heterozygous for *rSPL13* for comparison. We found that at day 8 when petioles are just about to develop in Col-0, *AN3::GUS* was localized to the proliferative region (Fig. 7A). At day10 when both the leaf blade and leaf petiole had elongated in Col-0, *AN3::GUS* was detected in the whole petiole and the proximal region of the blade, at much lower levels than its levels at day 8. At day 12, *AN3::GUS* could no longer be detected in leaf 1 and 2 of Col-0. The fifth leaf of Col-0 is normally an adult leaf in LD conditions and *AN3::GUS* could be detected in the petiole and the proximal region of the blade when its overall length was the same as its first leaf (Fig. 7A, inset). Petioles were not visible in 8-day-old *bop1/2* nor *rSPL13* plants, and *AN3::GUS* was more widely detected in their blades than Col-0 at this stage, with a higher concentration in the proximal region of the blade. Petioles were just visible in 10-day-old *bop1/2* and *rSPL13* plants, and their *AN3::GUS* expression was persistently detected in the proliferation zone at higher levels than its expression in Col-0 at day 10. At day 13 and day14, when leaf 1 and 2 of *bop1/2* and *rSPL13* plants were developed into the same length as Col-0 leaf 1 and 2, respectively, *AN3::GUS* could still be detected in the proximal region of the blade (arrows), indicating that cell proliferation persisted longer in the blades of *bop1/2* and *rSPL13* plants than in Col-0. These results indicate that the establishment of the proliferative region is delayed in *bop1/2* and *rSPL13* plants and the more elongated lamina in them may be a result of prolonged AN3 activity in their blade.

## Discussion

Up-regulation of miR156-targeted SPLs resulted in changes of heteroblastic traits in vegetative leaves (Wu *et al.*, 2009; Xu *et al.*, 2016b; He *et al.*, 2018). Lamina shape is one of the heteroblastic traits between juvenile leaves and adult leaves in Arabidopsis. Here, we show that a more elongated lamina in Arabidopsis adult leaves is caused by down-regulation of *BOP1/2*. SPL9 and SPL13 directly repress *BOP1/2* to delay petiole development, resulting in more blade outgrowth and more elongated lamina.

### The shape of the lamina and abaxial trichome production are controlled by different mechanisms

Although the *bop1/2* double mutant produces abaxial trichomes slightly earlier than WT, its lamina is much more elongated than that of WT, suggesting that BOP1/2 have a larger role in controlling the lamina shape than in trichome production. Several lines of evidence suggest that trichome production and lamina shape are controlled by different mechanisms. Trichome production is largely controlled by the trichome initiation gene *GL1*, which is repressed by the miR172-targeted *TOE1/2* genes (Wang *et al.*, 2019; Xu *et al.*, 2019). miR156-targeted SPLs directly activate *MIR172b*, which represses *TOE1/2* genes (Wu *et al.*, 2009). In the *GL1* dominant mutant *gl1-D*, whose TOE1/2 binding site is mutated, abaxial trichome production is accelerated but the lamina shape is not significantly different from WT, suggesting that lamina shape and abaxial trichome production are controlled by different mechanisms (Xu *et al.*, 2019). Consistent with this, abaxial trichome production in the *35S::MIR172B 35S::MIR156A* double mutant is close to that of *35S::MIR172B* while the lamina shape in the double mutant is close to that of *35S::MIR156A* (Wu *et al.*, 2009). In the *bop1/2 spl* sextuple mutant, trichome production is closer to that of the *spl* sextuple mutant. However, its lamina shape is closer to that of the *bop1/2* double mutant (Fig. 5). Together, these results suggest that abaxial trichome production in leaves is mainly controlled by the miR172–TOE module, while lamina shape is controlled by BOP1/2 and other unknown SPL targets.

### SPL controls leaf development along the proximal–distal axis by repressing *BOP1/2*

Previous analysis showed that the establishment of a proliferative region at the junction between petiole and blade is essential for leaf development along the proximal–distal axis in Arabidopsis (Ichihashi *et al.*, 2011). A proliferative region is established when the petiole has just developed from the proximal region of a leaf. *AN3* is highly expressed in this proliferative region and its expression decreases as the leaf matures (Ichihashi *et al.*, 2011). The spatial and temporal examination of blade and petiole development in Col-0, *bop1/2*, and *rSPL13* plants suggested that leaf development along the proximal–distal axis is largely dependent on the occurrence of the petiole: when petiole development is significantly delayed in both the *bop1/2* double mutant and *rSPL13* plants, the establishment of the proliferative region is delayed in them, resulting in longer AN3 activity and more blade outgrowth than in Col-0 (Fig. 7). In WT juvenile leaves, where activities of miR156-targeted SPLs are low, *BOP1/2* are highly expressed, and the proliferative region is established early*. AN3* diminishes sooner in juvenile lamina, resulting in a rounder lamina. In adult leaves, where activities miR156-targeted SPLs are increased, *BOP1/2* are down-regulated, and the establishment of the proliferative region is delayed. AN3 activity then persists longer in adult leaves than in juvenile leaves, resulting in a more elongated lamina (Fig. 7B). Leaf 1 and 2 in *rSPL13* plants and *bop1/2* are bigger than leaf 1 and 2 in Col-0, and it is quite possible that the increase in size is caused by down-regulation of *BOP1/2*. It is, however, not clear if this is connected to the activity of AN3, as expression of *AN3* is not expanded laterally in *rSPL13* plants and *bop1/2* leaves.

### Heteroblastic trait in Arabidopsis and rice

In Arabidopsis, the proximal region of a leaf develops as a petiole, and the distal region develops as a blade. In rice, the proximal region of a leaf develops as a sheath, and the distal region develops as a blade (Ichihashi *et al.*, 2011; Toriba *et al.*, 2019). Although the petiole and sheath develop at different stages in Arabidopsis and rice leaves, their development is controlled by similar genes. Sheath development is severely suppressed in the rice *osbop1/2/3* triple mutant, and petiole development is severely suppressed in the Arabidopsis *bop1/2* double mutant (Hepworth *et al.*, 2005; Norberg *et al.*, 2005; Jun *et al.*, 2010; Toriba *et al.*, 2019), suggesting conserved roles for BOP in monocots and dicots. Studies in rice found that miR156-targeted SPLs repress Os*BOP1/2/3* expression to control sheath development. Here, we found that miR156-targeted SPL9 and SPL13 repress *BOP1/2* directly in leaves to control petiole and blade development (Figs 4, 6).

The lamina shape is a heteroblastic trait in Arabidopsis, while the relative sheath length is a heteroblastic trait in rice (Wu *et al.*, 2009; Yang *et al.*, 2012; Xu *et al.*, 2016a, b; Toriba *et al.*, 2019; Cheng *et al.*, 2021). Here, our data showed that the lamina shape is correlated to the relative petiole length in juvenile leaves and adult leaves (Fig. 2). Together, studies in Arabidopsis and rice suggest that changes in the three ratios blade length: blade width, petiole length: whole leaf length, and leaf sheath: blade are caused by disrupted development at the proximal region of leaves, petiole, or sheath. This suggests that the development of the proximal region of a leaf may be a key factor for heteroblastic traits given the conserved roles of BOP1/2 and SPLs in higher plants.

## Supplementary data

The following supplementary data are available at *JXB* online.

Fig. S1. Plants ectopically expressing SPL13 mimic *bop1 bop2* double mutant. 

Fig. S2. BOP1 and BOP2 act redundantly to promote petiole development and suppress blade outgrowth.

Fig. S3. BOP1/2 promote while SPL9 and SPL13 suppress petiole development.

Fig. S4. Ectopic expression of *GFP-rSPL9 spl9/13* accelerated abaxial trichome production in both LDs and SDs.

Fig. S5. RT-qPCR analysis of gene expression normalized to *EIF4A1*. 

Table S1. Primers used in this study.

erad017_suppl_Supplementary_Material

## Data Availability

All data generated or analysed during this study are included in this published article and its supplementary data published online.
