# Supplementary material for: SQUAMOSA PROMOTER BINDING PROTEIN-LIKE 9 and 13 repress BLADE-ON-PETIOLE 1 and 2 directly to promote adult leaf morphology in Arabidopsis
Source: J Exp Bot. 2023 Jan 11;74(6):1926–39. doi: 10.1093/jxb/erad017 (PMC10049914; doi:10.1093/jxb/erad017)
Supplement: erad017_suppl_Supplementary_Material [file erad017_suppl_supplementary_material.pdf]

Figure S1

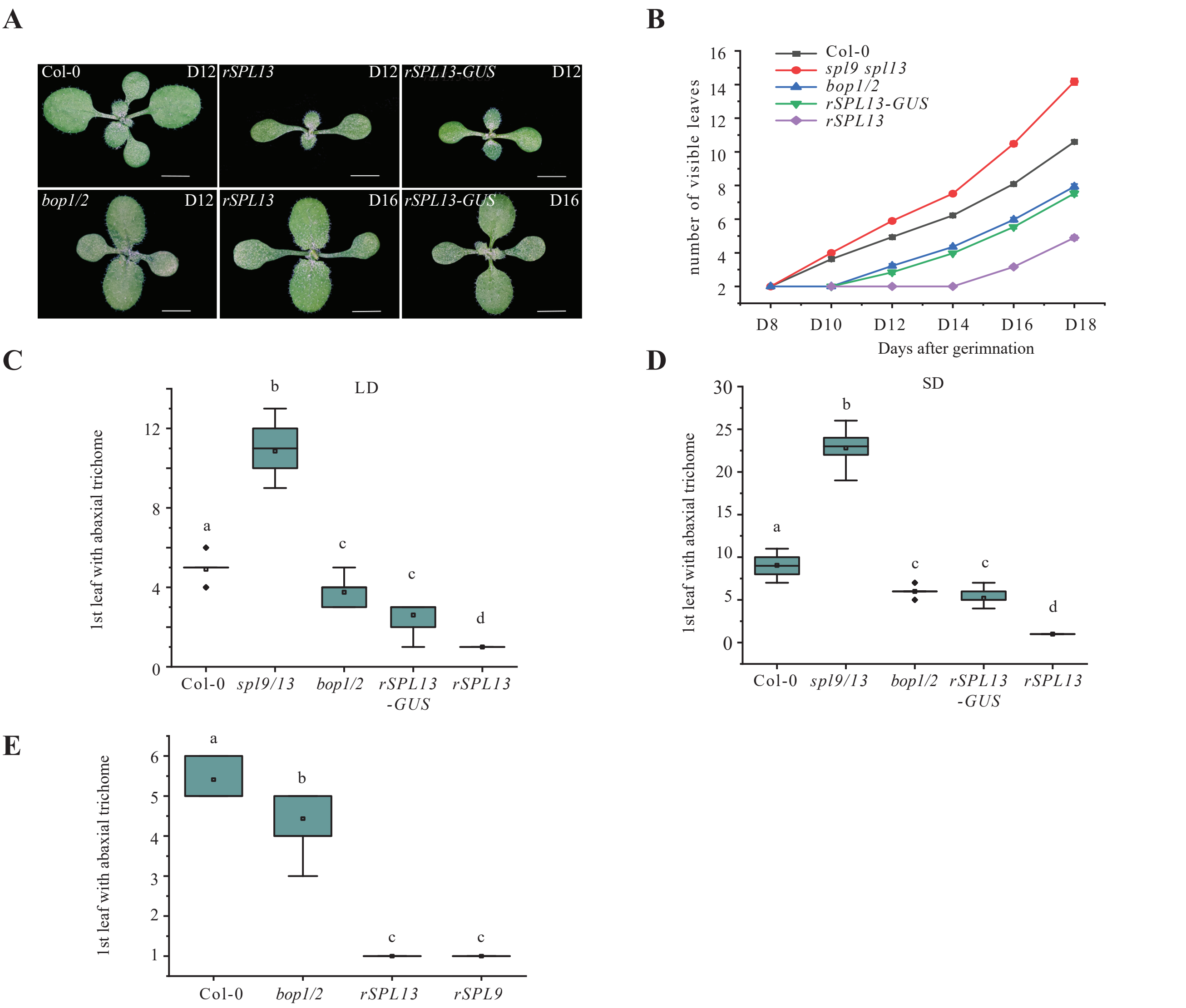

Fig. S1. Plants ectopically expressing SPL13 mimic *bop1 bop2* double mutant. (A) Rosettes of Col-0, *bop1/2* and *rSPL13* plants in LD conditions. Bars=3mm. (B) Leaf initiation is accelerated in *spl9/13* double mutant and delayed in *bop1/2* and *rSPL13* plant. (C-D) Vegetative phase change in Col-0, *spl9/13*, *bop1/2*, *rSPL13-GUS* plant and *rSPL13* plant in LDs (C) and SDs (D). (E) Vegetative phase change in Col-0, *bop1/2*, *rSPL9* and *rSPL13* plants in LDs. Different letters indicate significantly different groups, P<0.001, one-way ANOVA.

Figure S2

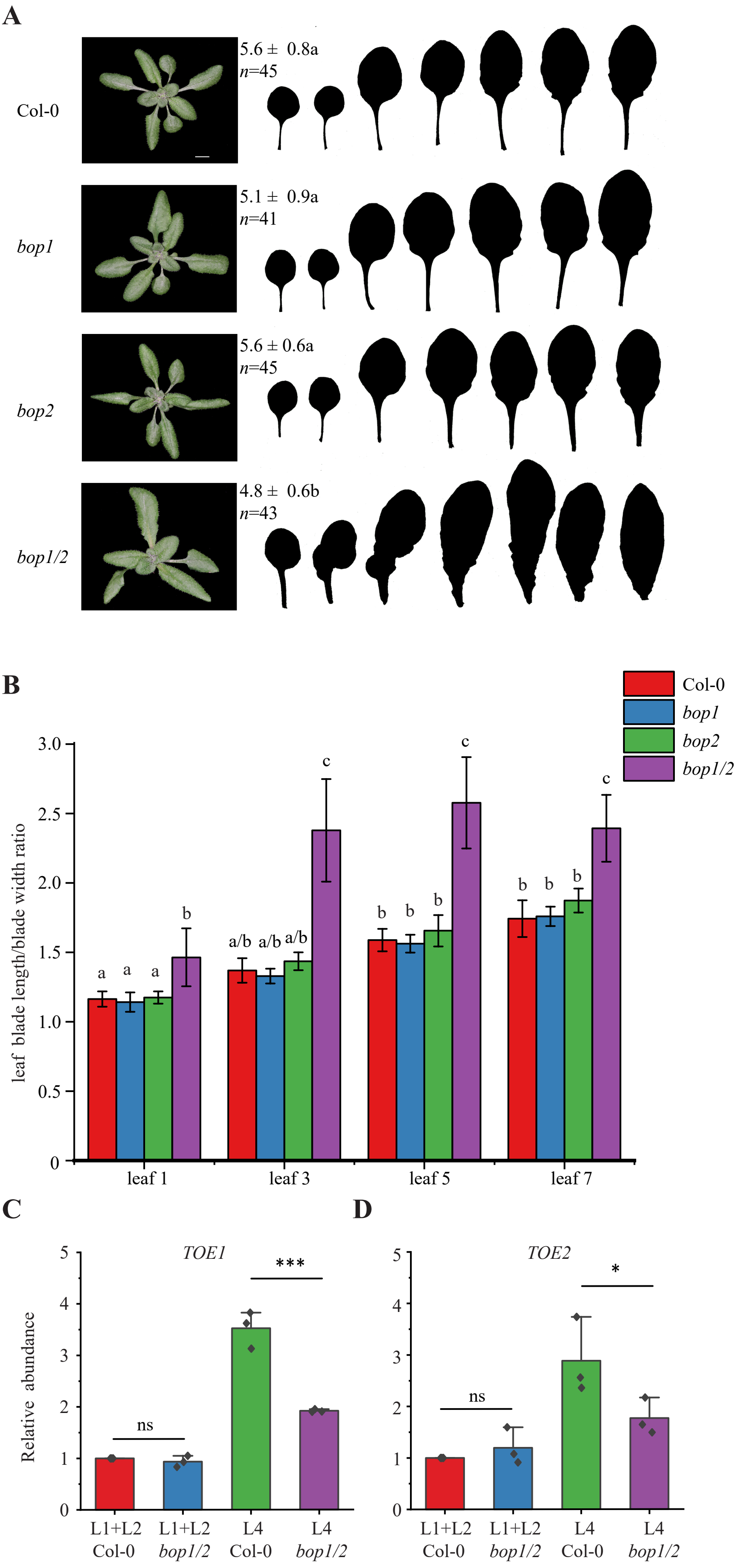

Fig. S2. BOP1 and BOP2 act redundantly to promote petiole development and suppress blade outgrowth. (A) Rosettes and heteroblasty of Col-0, *bop1* single mutant, *bop2* single mutant and *bop1/2* double mutant. Numbers indicate the first leaf with abaxial trichome in each genotype. Shared letters denote not significantly different groups, p>0.05, one-way ANOVA. (B) Leaf blade length:width ratio in leaf 1, leaf 3, leaf 5, and leaf 7 of Col-0, *bop1*, *bop2* and *bop1/2*. The blade length:width ratio in each leaf of *bop1/2* double mutant is significantly higher than the corresponding leaves in Col-0, *bop1* or *bop2*, while the ratio in *bop1* or *bop2* single mutants are not higher than Col-0 in each leaf. Shared letters above bars denote not significantly different groups, different letters above bars denote significantly different groups, p<0.05, two-way ANOVA. (C-D) Transcripts of *TOE1* (C) and *TOE2* (D) in leaf 1 and 2 (L1+L2) and leaf 4 (L4) of Col-0 and *bop1/2* double mutant. The levels of *TOE1* or *TOE2* in Col-0 L1+L2 were set to be 1. Values are from three independent biological replicates (diamonds). ***p<0.001, * p<0.05, ns, not significant, one-way ANOVA.

Figure S3

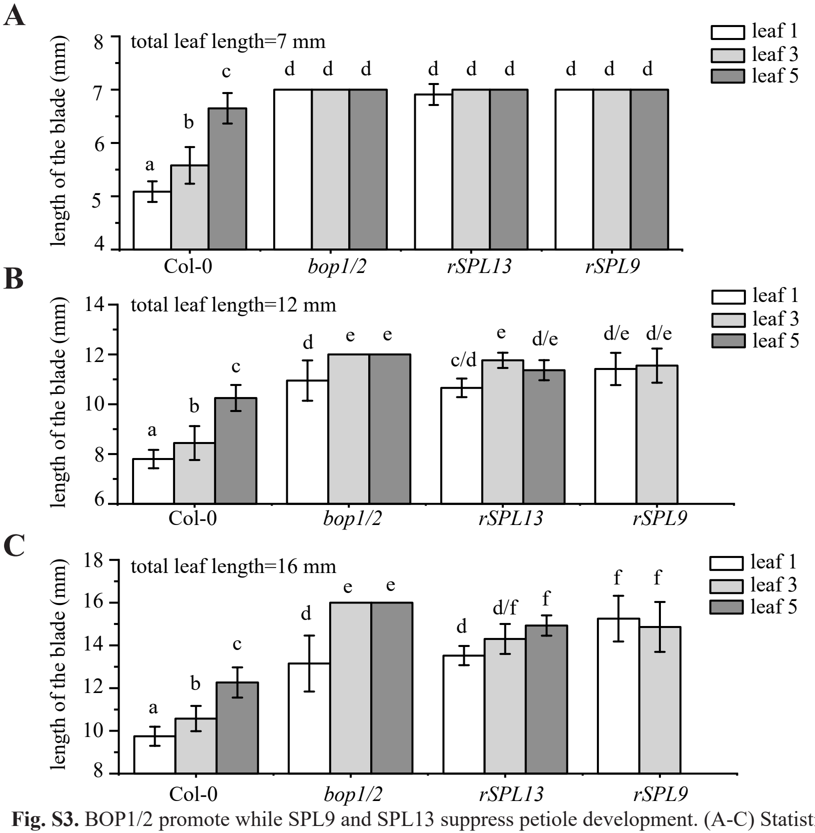

Fig. S3. BOP1/2 promote while SPL9 and SPL13 suppress petiole development. (A-C) Statistical analysis of the length of the blade when the leaf is 7 mm long (A), 12 mm long (B), and 16 mm long (C).

Figure S4

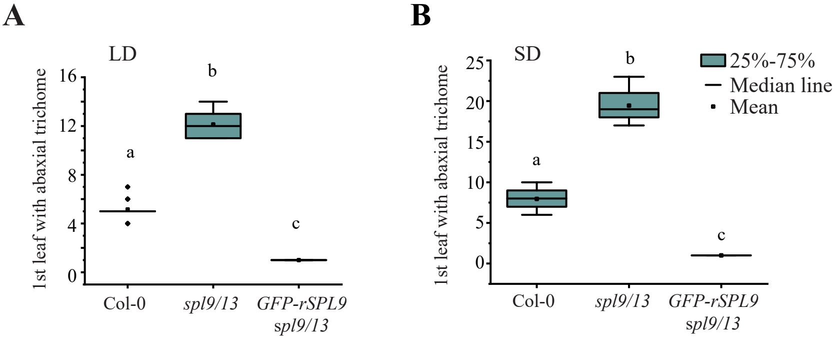

Fig. S4. Ectopic expression of *GFP-rSPL9 spl9/13* accelerated abaxial trichome production in both LDs (A) and SDs (B). Different letters above each boxes indicate significantly different groups, $p<0.001$, one-way ANOVA.

Figure S5

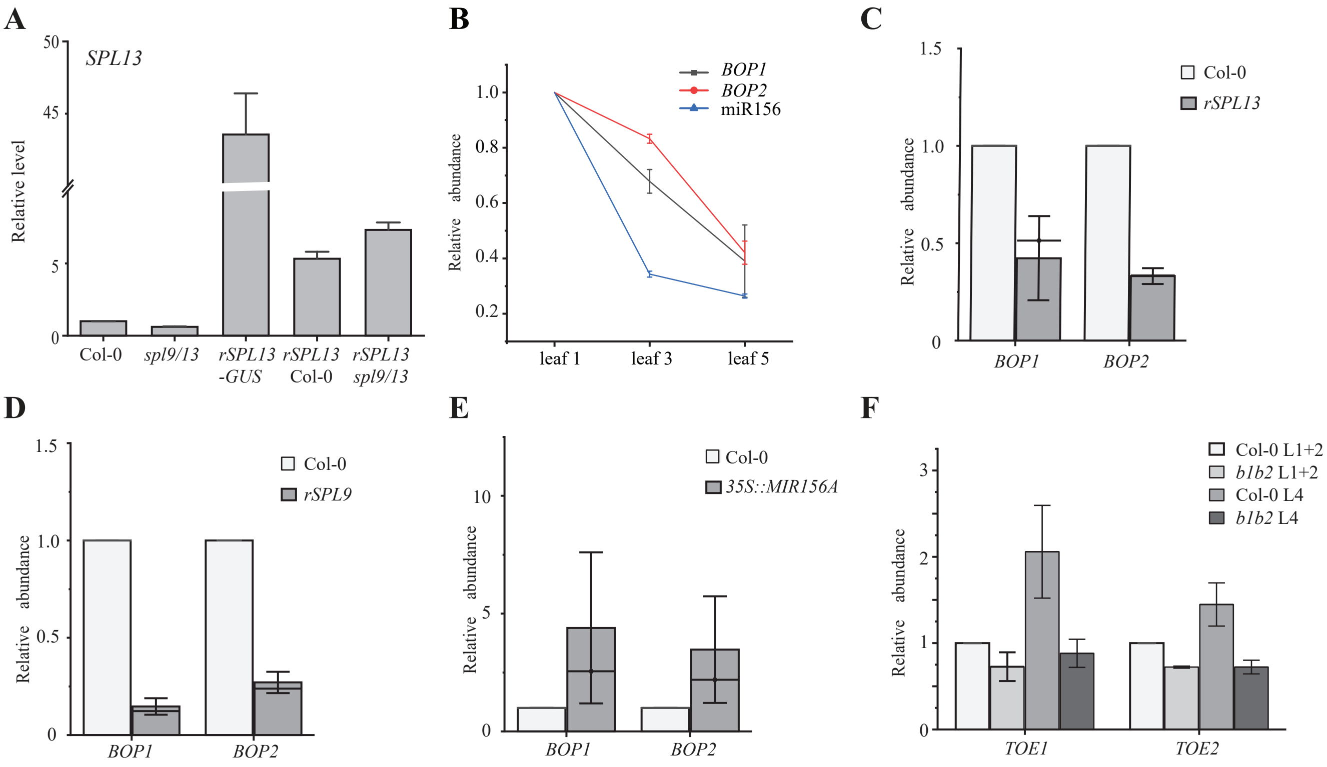

Fig. S5. RT-qPCR analysis of gene expression normalized to *EIF4A1*. (A) RT-qPCR analysis of *SPL13* in diferent plants. (B) RT-qPCR analysis of *BOP1*, *BOP2* and miR156 in leaf 1, leaf 3, and leaf5. (C-E) RT-qPCR analysis of *BOP1* and *BOP2* in Col and *rSPL13* plant (C), *rSPL9* plant (D), and *35S::MIR156A* (E). (F) RT-qPCR analysis of *TOE1* and *TOE2* in Col-0 and *bop1/2* leaves.

Supplementary Table 1. Primers used in this study.

primers for RT-qPCR	5'-3'
BOP1 qPCR F	GTATCAAAGAAATCAACAAAGGAG
BOP1 qPCR R	CGTTGATTAAGAGGTTTAGGTAAT
BOP2 qPCR F	GGAAGGTATGAGTCGGCATC
BOP2 qPCR R	TGCATGCCCCTCTTCTTAAT
SPL13 qPCR F	GGGTTTTCAAGGTAGCAAATTGCT
SPL13 qPCR R	ACCAACAACATAGCTCTGGCTCTG
TOE1 qPCR F	ACCGGTAATGCGCCAAAGCAAA
TOE1 qPCR R	ACCGGCTTTCCCATGTATTCGT
TOE2 qPCR F	TGCCCTTCCTTCTGCGTTCTTT
TOE2 qPCR R	ACTGATCATGCCCTTGCCATGT
ACT2 F	GCACCCTGTTCTTCTTACCG
ACT2 R	AACCCTCGTAGATTGGCACA
EIF4A1-F	ACAGTTTGATGCACGTCAGTTTG
EIF4A1-R	TCTCAAAACCATAAGCATAAATACC
Primers for ChIP	5'-3'
BOP1 ChIP F1	TTAAGTTAGTACAGTGGAGACTTT
BOP1 ChIP R1	CATATGCATGCGTATTAAATTGCTA
BOP1 ChIP F2	CTATCATTTTACTGCACAATCTTTC
BOP1 ChIP R2	ATGCTAATAAAATGAGGACTACTTC
BOP1 ChIP F3	AATACTACAAAACTCCAAATGCTAG
BOP1 ChIP R3	GAGAGAGACTATAGTGTTTAGAAGA
BOP2 ChIP F1	ACAACATGCAAATCAGTAAGAAA
BOP2 ChIP R1	TCCTAGTAAGATTAGCCTAAAAT
BOP2 ChIP F2	TTCTCTCTCTCTCTTTCTTTCTTT
BOP2 ChIP R2	TTTATTGATCTTGTTGGCTATGAAG
BOP2 ChIP F3	AACCTACTAATCAACGGTCAAG
BOP2 ChIP R3	ATGTTGGGTCGGGTCTATAC
TA3 F	CTGCGTGGAAGTCTGTCAAA
TA3 R	CTATGCCACAGGGCAGTTTT
Primers for making AN3 promoter	5'-3'
Bpil-AN3-Pro-F1	TTGAAGACAAGGAGAATTGTTTTGGATATCTCGAAGTTAT
Bpil-AN3-Pro-R1	TTGAAGACAATCTCTAAAGTTTTTGAAATGCTTTTGT
Bpil-AN3-Pro-F2	TTGAAGACAAGAGATCCTTTGGTTGAGAATCATCAATATT

Bpil-AN3-Pro-R2	TTGAAGACAATCCTCTTATGCGGATTAGATTTGGCAT
Bpil-AN3-Pro-F3	TTGAAGACAAAGGACTAAGTCACAACATAATTCAGTAATTT
Bpil-AN3-Pro-R3	TTGAAGACAAAACCTTTACATATATACTACTACTTATCAATATGT
Bpil-AN3-Pro-F4	TTGAAGACAAGGTTTCACGTCTCTATAATAAAGTATGACT
Bpil-AN3-Pro-R4	TTGAAGACAACATTCCAGCCATCATGGGCTGCATCT
primers for making SPL13 constructs	5'-3'
SPL13 pro F1	TTGGTCTCAACATGGAGTTTTTTGTTAACCGGAAAATTGCTTTC
SPL13 pro R1	TTGGTCTCAGTCCTCCATGTCTTTAAAAGTTTTGCAG
SPL13 pro F2	TTGGTCTCAGGACTTTTATACGATTCTCCCTGATAA
SPL13 pro R2	TTGGTCTCAACAACATTCAAAGATTGTGCTTTTTTTTCTTCTCTC
SPL13 CD F1	TTGAAGACAAAATGGACTGGAATTTCAAACTTAGCTCTGGTT
SPL13 CD R1	TTGGTCTCATCGCCTTCGATTATGTCCATCAAGACG
SPL13 CD F2	TTGGTCTCAGCGACGGAAGCCGCAGCCTGAACATAT
SPL13 CD R2	TTGGTCTCAACAACGAACCCTCCCAATGAAACGGGAATGTCTGCGGGAGACGG
SPL13 CD R4	TTGAAGACAAAAGCCTACTCCCAATGAAACGGGAATGTCTGCGGGAGACGG
SPL13 3UF1	TTGGTCTCAACATGCTTTAGAAGAAGAAGTAGGTAGATAGATAGAAT
SPL13 3UR1	TTGGTCTCAAAGGCTGTACAAGATTATGAGAAGAATTGGA
SPL13 3UF2	TTGGTCTCACCTTCTTCTTCTCTCCAATCAAACTTCAAAC
SPL13 3UR2	TTGGTCTCAACAAAGCGGCTCAGAGAAGAGATAGTGATGTTAATTCT
primers for making SPL9 constructs	5'-3'
SPL9 proF1	TTGGTCTCAACATGGAGGGTTTATCACATTTGAGCGTCCAAAGATATGGTCCCTG
SPL9 proR1	TTGGTCTCACTGGGACCAGGGAGCAATATTCAGACTCCCC
SPL9 proF2	TTGGTCTCACCAGATAGATGCTTTCAAAATTTAATACTAC
SPL9 proR2	TTGGTCTCATCTGCGTGAGTAGAGGATAACAACAAATGTATGGTCCCT
SPL9 proF3	TTGGTCTCACAGACAACCAGTCTAACGTTATTGCATAAG
SPL9 proR3	TTGGTCTCAACAACATTGTTGGTTTCCTCTTACTCAGACAGAAAGGG
SPL9 CDF1	TTGGTCTCAACATAATGGAGATGGGTTCCAACTCGGGTCCGGGTCAT
SPL9 CDR1	TTGGTCTCAAGAGGACCCGGATCCACCACCACCGTCCTCG
SPL9 CDF2	TTGGTCTCACTCTTCCTCAGGTGGTCGTTCAAACAGACG
SPL9 CDR2	TTGGTCTCAACAAAAGCTCAGAGGGACCAGTTGGTATGGTGAGAAGAAG
SPL9 3U F1	TTGGTCTCAACATGCTTCTTGTCTTTGCATCAGAGAATCTTCTTAC
SPL9 3U R1	TTGGTCTCAGTCCTCTGATGAGTATATATGGTTCAAGTTT
SPL9 3U F2	TTGGTCTCAGGACAACTTAACTATGGCAGCTTTTCAAAT
SPL9 3U R2	TTGGTCTCAGTCCCTATCATCGTCTTACTTTTACTGTTTC
SPL9 3U F3	TTGGTCTCAGGACCTTTCTTCCTAAAGATCTCTCATCATC

SPL9 3U R3	TTGGTCTCAACAAAGCGTTCTTCAGGAGACGAGTCAGTAGAGCCAGAG
Primers for genotyping	5'-3'
spl2-1 LP	CTTTAAACCGAGAACCGGATC (T-DNA: LBb1)
spl2-1 RP	TGAATAGTGGAAGAGAGAAAGCTTC
spl9-4 LP	TGGTTCCTCCACTGAGTCATC (T-DNA: LB3)
spl9-4 RP	GCTCATTATGACCAGCGAGTC
spl10 LP	AGGACAAACGATGCAATCTTG (deletion)
spl10 RP	TTTTCTTCCGAGCAACAACAG
spl 11 LP	GGACGAGGTTTTTATCATAGGTTTGG (T-DNA: Flag LB4)
spl11-1 RP	GTTGCATTCTCTTTAGATTTTACTGTA
spl13 LP	GCTCGAGTTCAAAGAGAACAAG
spl13 RP	CAATCTTACCTGCTGCATTGTC (dCAPS digest with Sal I)
spl15 LP	TGTTGGTGTCTGAAGTTGCTG (T-DNA: LBb1)
Spl15 RP	TCCACCGAGTCTTCTTCACTC
bop1-3 LP	GCACAATCTTTCGACTTCATCACC (T-DNA: LBb1)
bop1-3 RP	CGTACCCTTTGATTTTAGTATGCTG
bop2-1 LP	AAAGAGAGAACCTGGGTGAGC (T-DNA: LBb1)
bop2-1 RP	ATTTGGCCCACCTTTGTATTC
LBb1	ATTTTGCCGATTTCGGAAC
LB3	TAGCATCTGAATTTCATAACCAATCTCGATACAC
Flag LB4	TGTGCCAGGTGCCCACGGAATAGT
